# Skill-mix in preventive dental practice - will it help address need in the future?

**DOI:** 10.1186/1472-6831-15-S1-S10

**Published:** 2015-09-15

**Authors:** Paul Brocklehurst, Richard Macey

**Affiliations:** 1 NWORTH, Y Wern, The Normal Site, Bangor University, Holyhead Road, Gwynedd, UK; 2 School of Dentistry, University of Manchester, Oxford Road, Manchester, M13 9PL, UK

## Abstract

**Background:**

Population health needs are changing. The levels of dental caries and periodontal disease across the population as a whole is falling. The proportion of adults with a functional dentition in many developed countries has increased substantially and edentulous rates have dropped to some of their lowest levels. Despite this, a pronounced social gradient still exists, many adults do not attend dental services regularly and disease in young children remains intransigent amongst the poorest. New challenges are emerging too as the growing number of older people, above sixty-five years of age, retain their teeth.

**Methods:**

Ensuring “the right number of people with the right skills are in the right place at the right time to provide the right services to the right people” is critical for future dental service provision, both to meet the new challenges ahead and to ensure future services are cost-effective, efficient and reduce health-inequalities. Greater use of “skill-mix” models could have a substantial role in the future, as dentistry moves from a “cure” to a “care” culture.

**Discussion:**

The provision of dental services in many countries currently adopts a “one-size-fits-all”, where the dentist is the main care-giver and the emphasis is on intervention. As needs change in the future, the whole of the dental team should be utilised to deliver primary, secondary and tertiary prevention in an integrated model. Growing evidence suggests that other members of the dental team are effective in providing care, but introducing this paradigm shift is not without its challenges. The provision of incentives within funding systems and social acceptability are amongst the key determinants in producing a service that is responsive to need, improves access and delivers equity.

## Introduction

Designing the most appropriate dental workforce for the future is critical to ensure “the right number of people with the right skills are in the right place at the right time to provide the right services to the right people” [1]. The aim of this paper is to explore how the greater use of “skill-mix” could ensure that the dental workforce is “fit-for-purpose” and meet the challenges ahead. “Skill-mix” is a term that is used to describe a model of care where the whole of the clinical team is utilized in delivering service activity [2-4]. It can be further sub-divided into role-substitution and role-supplementation. The former is where different members of the dental team undertake clinical tasks instead of a dentist, whilst the latter is where team members augment the activity of a dentist. It will be argued in this paper that both models could have a substantial role in the future, as dentistry moves from a “cure” to a “care” culture.

### Changing population need

Population health needs are changing. In the most recent epidemiological survey undertaken in the United Kingdom (UK), the levels of dental caries and periodontal disease in adults both fell and are predicted to continue to fall into the future [5,6]. In addition, the proportion of adults with a functional dentition had increased and over ninety percent of young adults are expected to have more than twenty-one teeth in ten year's time. Edentulous rates have also dropped to their lowest levels (six percent). However, significant variations in oral health across different geographic regions continue to exist, with a pronounced social gradient [6]. The levels of disease in young children from deprived areas remains intransigent; for example, the rate and severity of caries experience found in five year olds nationally are found in three year olds from Greater Manchester [7]. New challenges are emerging too. Fifty percent of the population in the UK will be over fifty years of age by 2050 and a quarter will be “older people” (over sixty-five) [8,9]. Four percent of all “older people” will live in care homes and most will have their own teeth [5,10]. Health inequalities also persist in access to services in the UK. Approximately, forty-five percent of the adult population with much of the active primary disease do not access care routinely. In contrast, over sixty percent of the National Health Service activity for dentistry is in delivering routine “check-ups” for regular attenders with limited comparable disease [11]. As the proportion of older people increase, inequalities in access to services is likely to be exacerbated further.

The UK is not alone in these changing patterns of population need. Similar patterns of changing need are occurring across all the developed countries [12]. As a result, it is important to develop a dental workforce *a priori* that is capable of responding to these changes ahead. This is likely to require greater stratification of service provision for the different population groups, prevention strategies based on the best available evidence and a re-orientation of dental practices from a “cure” to a “care” culture [12]. There will be a need to prevent and manage dental disease in existing adult and child patients who attend their dental practice on a regular basis. This will increasingly include those patients with limited or little disease experience, alongside those who have experienced complex restorative treatment in the past and require ongoing maintenance. However, there will also be a need to target those groups who don't or can't access care.

### Role-substitution in dentistry

Role-substitution by non-dentist oral health-care workers, like dental hygienists and hygiene-therapists, is long established in a number of countries [13,14]. Models of use varies considerably, as does the terminology. The remainder of this paper uses the term hygiene-therapists (H-Ts) to describe this group of professionals who can undertake direct restorative procedures on patients, including simple conservation and periodontal treatment. Within Europe the dentist to H-T ratio was 18:1 in 1985, but this has risen to 11:1 in 2010 [15]. However, “there are still only a handful (of) countries where the hygienist numbers are great enough to make a significant difference to the delivery of oral health care” [15]. Outside of Europe this ratio is 1.5:1, with a population: H-T ratio of 2000:1 [15].

Proponents argue that role-substitution in dentistry has the potential to increase the efficiency and effectiveness of service provision; releasing resources to improve access to care and reducing oral health inequalities [16,17]. In a review of the literature, Nash concluded that “access to basic dental care will not be available without the utilisation of dental therapists in the workforce” [13], whilst Johnson argues for a paradigm shift using H-Ts to shift the culture “from treatment to prevention, wellness and self-care” [18]. In medicine, there has been a dramatic shift in the use of role-substitution [3,4] and evidence has shown that nurses are as effective as doctors for the more common and simpler aspects of care [19-21].

There are two principle models of role-substitution: integrated and independent practice. The former describes the use of H-Ts within an existing team that is led by a dentist, whereas the latter describes a model where H-Ts practice independently. Sweden and The Netherlands where among the first countries to allow independent practice for dental-hygienists, which was legalised in 1964 and 1978 respectively. The legal framework in The Netherlands permits “functional independence” that allows dental-hygienists to practice mainly on their own. However, it also requires close co-operation between the dental hygienist and the dentist for the diagnosis and management of dental caries. Dentists are also required to prescribe and interpret radiographs. Finland, Denmark and Norway have allowed independence since 1994, 1996 and 2001. Similar practices are found in Switzerland, which started in 1997 and in Italy, dental-hygienists have been able to work as independent practitioners since 1999. In the United States and Canada, dental hygienists are a growing profession and can practice with varying degrees of independence in California, Colorado, Montana, Nebraska, New Mexico, Oregon, Washington British Columbia, Alberta, Saskatchewan and Manitoba.

Tasmania has a liberal regulatory model where dental-hygienists and dental-therapists practice independently and can own their own practices. The legal framework is identical for both dentists and H-Ts, but the latter group requires a documented agreement with a dentist in order for them to seek consultation and provide referral. Dental therapists are also considered to be independent in New Zealand, although they are not able to treat adults. In Samoa and Singapore, dental-therapists must work under the supervision of a dentist. Fiji has allowed dental-therapists to assume independent responsibility for managing clinics since 1985 and dental-therapists have been allowed to practice independently in South Africa since 1994.

In the UK during 2013, the legal framework was changed to allow patients to directly access H-Ts, without the need for the patient to first see the dentist. Following this the Scope of Practice was expanded and H-Ts are now allowed to carry out a clinical examination, diagnose and undertake a treatment plan within their competency. As such, appropriately trained H-Ts can now administer local analgesia, extract deciduous teeth and place direct restorations in children and adults, alongside the provision of primary prevention.

### Mapping the future dental work-force onto population need

**Figure 1:**
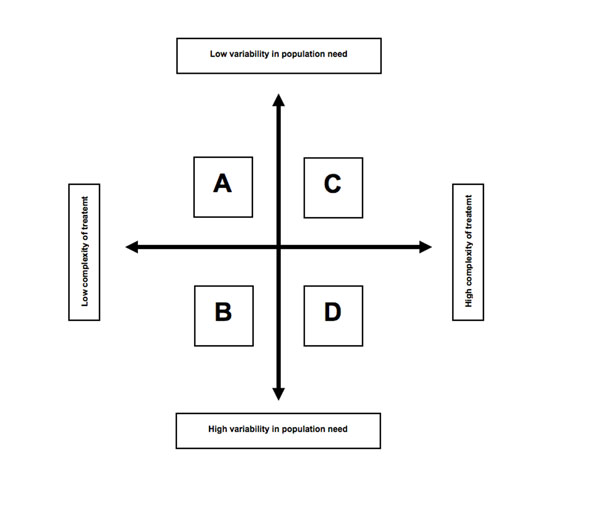
Stratification of the future dental workforce

To meet the challenges ahead in dentistry, it would seem sensible to map the future dental workforce onto future population need. Figure 1 examines this change based on two principle dimensions: variability in population need and the complexity of treatment required to meet this need. In terms of the variation of population need, dentistry is different to medicine. Unlike the wide range of diseases that present to doctors, there are principally two diseases that constitute most of the burden for dentistry: dental caries and periodontal disease. Although tooth wear is becoming an increasing problem, oral cancer is rare and skeletal and dental anomalies are constant at a population level. As highlighted above, the projected epidemiological trends suggest that there will be an increase in healthy children and adults in the future, who are predominantly disease free. However, there will remain an aging cohort of patients who have experienced a large amount of restorative care, whose presenting needs are likely to be ongoing and complex. There will also be an increasingly aging population, whose needs will vary and a group who do not access care that commonly present with primary disease.

In terms of complexity of treatment, dentistry has progressed substantially over the past fifty years due to the impact of fluoride and the major advances in technology that have occurred [2]. High strength ceramic and composite restorative materials have revolutionised the placement of indirect and direct restorations and titanium implants are beginning to be common place. Looking towards the future, this is set to continue but the changing patterns of population need is likely to mean that dentistry shifts from a “cure” to a “care” culture, with the bulk of the management of patients being based on prevention in two to three decades time. There will remain those regular patients who have experienced high levels of restorative treatment and who will continue to require complex management, but as this group age further and lose their independence, their care is likely to become increasingly based on preventing future disease and managing function [22]. For the younger cohorts of patients who present to their dental practitioner, much of their care will be increasingly managed by prevention and the placement of simple restorations. For those that don't attend regularly, the key is stopping the expression of the disease and arguably the most effective strategy is a population approach, rather than a practice or risk based strategy [23].

The evidence from the epidemiological data suggests that the bulk of future clinical activity will increasingly be in Area A (Figure 1) in the future i.e. management of those patients with limited or little disease. Although it is likely that some restorations may be required, the bulk of the activity will be preventive. Prevention is also advocated for young children and those that are aging [22]. Many, if not all of these activities could be undertaken by H-Ts at a practice level. Area B represents those of the population with complex dental histories, which would require more complex diagnostic decisions to be made. These activities would need to be undertaken by a clinician with the diagnostic skills pursuant to a primary care dentist. However, given the low complexity of their management, treatment could then be undertaken at a practice level by H-Ts. Alternatively, it could be delivered by a dentist and supplemented by H-Ts to deliver prevention and maintenance. Area C and D reflect those of the population who require more complex treatment. The management of these patients should be undertaken by dentists and by referral onto appropriate specialists, particularly in Area D. However, care could again be supplemented by H-Ts to deliver prevention and maintenance.

The ramifications for stratifying the workforce according to population need using the model in Figure 1 are challenging, not least to professional boundaries. However, it is an ethical imperative for health care planners to provide both effective and efficient services and with population need falling, the case for greater role-substitution and supplementation is likely to increase. Using the most expensive resource (the dentist) to manage disease that could be managed by lower paid health care workers is likely to become increasingly less attractive, particularly in those countries who have a public-funded service and are mandated to provide dental care for their citizens [23,24]. However, an equal imperative is to ensure that the patient receives an appropriate level of co-ordinated care and so access the “right skills […] in the right place at the right time” [1]. As such, an integrated rather than an independent model of role-substitution would appear to be the most appropriate, where dentists can oversee large teams of H-Ts and provide appropriate consultation and receive referrals for more complex care.

### Evidence for greater use of H-Ts

The evidence supporting the greater use of H-Ts is emerging. Prevention can be divided into primary, secondary and tertiary strategies. Dental hygienists and hygiene-therapists have for a long time been considered to be the most useful health care worker to deliver the former. In Galloway's systematic review, they were found to be better than dentists at oral health promotion and in the most recent Cochrane systematic review, survival rates of resin fissure sealants were similar over various time periods [25,26]. However, the quality of the evidence from both reviews was considered to be limited due to the lack of experimental designs [25,26].

There is also evidence of the ability of H-Ts to screen for disease; a secondary prevention strategy. There are many definitions of screening, but all imply an ongoing, structured healthcare intervention designed to detect disease at an asymptomatic stage [27,28]. Screening is distinct from an examination or diagnosis as its purpose is to simply determine the probable presence or absence of disease in asymptomatic individuals.

Given the increasing numbers of healthy patients attending practice on a regular basis, deploying H-Ts for this task could allow for the routine surveillance and monitoring of low risk and regularly attending patients. This would map onto Area A of Figure 1. There is evidence for the use of H-Ts to detect caries in both the primary and secondary dentition [29-33]. H-Ts have also been shown able to screen for oral cancer. The only randomized controlled trial that has examined screening for oral cancer used allied health providers, not dentists, to undertake the screen and judgement under uncertainty favours safety [34-36].

In an ongoing “National Institute of Health Research” funded programme of work, H-Ts have been assessed on their ability to screen for the common dental diseases and for oral cancer [37]. When compared against a dentist as the gold standard, dental-hygienists were found to be able to detect 82% of the patients who the primary care dentist thought had dental caries and 85% of those that were deemed to be free of the disease. They were also able to detect 89% of cases that the dentist thought had periodontal disease and 75% of those that were deemed to be healthy [37,38]. These figures for a screening test are very high compared to agreed standards and the study has a high number of participants (n = 1,899). Equally, in a parallel study, there was no difference in the sensitivity and specificity between H-Ts and primary care dentists in the detection of oral cancer and potentially malignant disorders (77% and 76% compared to 71% and 68% respectively) [38]. H-Ts missed fewer frank malignancies.

In terms of tertiary prevention, treatment outcomes for atraumatic restorative techniques were similar across a number of criteria for single surface (small) and multi-surface (large) restorations between H-Ts and dentists [26]. In addition, a recent literature review found that restorations placed by H-Ts and dentists were considered to be of equal quality [26,39].

The evidence would therefore suggest that H-Ts could be used to supplement or substitute care by a dentist and play a key role in the workforce of the future. Again, this is contentious, but it could also mean that all of the dental team get up-skilled; H-Ts taking on the more routine tasks (Area A and B), with dentists becoming more highly skilled in their diagnostic capability and management strategies (Areas B to D). This is currently happening in the UK. Training pathways are just emerging to develop “Dentists with Enhanced Skills”, to take on more of the service activity that has traditionally been undertaken previously by Specialists and NHS Consultants (the type of activity that would lie in Area D). The effect of this will be to move much of the care into primary care and away from hospitals and acute settings. As population need changes, all sectors of the workforce will come under greater scrutiny to ensure health service planners maximise health for a given level and mix of resources [24]. Although dentistry has traditionally based future workforce projections on historic patterns of service provision, the profound changes to population need is likely to lead towards using a more stratified, preventative approach. A different needs-based planning model will be required [40].

### Levers for change

This all sounds good theoretically. However, if population need does change as predicted, what drivers will ensure the dental workforce align with the objectives of any given health system to address this change in need? There are many factors here, including challenges to autonomy, changing the primary care model from a “cure” to a “care” culture and ensuring that the dental estate is “fit-for-purpose”, but the predominant driver is likely to be financial [12].

**Figure 2:**
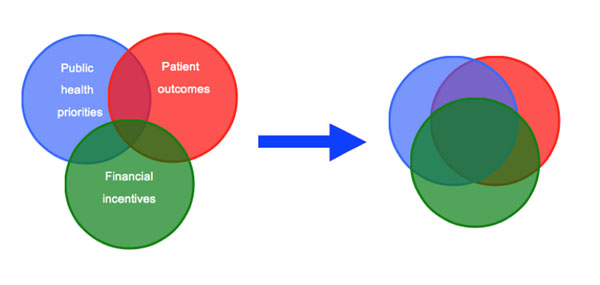
Aligning the work-force with population need to promote quality

Dentists are part of an altruistic profession, although much of their activity in primary care is driven by the “profit principle” to maintain the viability of their practices. As such, primary care dentists are acutely sensitive to incentives within the remuneration system [41-44]. Retrospective payment systems or “fee-for-item” commonly leads to over-treatment [41-45]. Conversely, prospective systems or “per-capita” payments often lead to under-treatment [45,46]. Where incentives promote preventive activity, primary care dentists tend to actively engage and shape their dental team accordingly, greatly increasing the use of role-supplementation and substitution [47,48]. The key principle then is to create a needs-based planning model *a priori* and then create incentives to ensure the workforce meets these needs, whilst improving patient outcomes (Figure 2). Creating greater alignment between these three important factors will then help drive up the quality of care received by the whole of the population; promoting an effective and efficient service that is based on equity and need, whilst improving access [47,49].

The final domain of quality is social acceptability [49]; will patients accept a different dental workforce and a change to their experience of care? Evidence from medicine would suggest that patients value role-substitution and supplementation, as H-Ts have more time to devote to the patient compared to doctors [3,4]. As population need changes and the culture in dentistry moves from one of “cure” to “care”, this is likely to become increasingly important, as the emphasis moves towards health promotion and primary and secondary prevention for regular attenders. H-Ts also could have a valuable role in reaching out to those in the population that currently don't access care, particularly for the young and old in the age distribution.

### Summary

With the predicted changes in population, there will be a pressing need to rethink how dentistry is delivered in the future, particularly in those countries who are mandated to provide state care. Many of those who regularly attend practices will increasingly require simple maintenance. As those who have experienced the bulk of the disease burden age further, prevention is also likely to become key. Access to services is likely to remain an important priority to reduce health inequalities for those that do not attend regularly, particularly in children and those that can no longer attend, due to poor mobility and a loss of independence. Role-substitution and supplementation has an important part to play for all of these predicted changes and challenges ahead. Within an integrated team, the dentist and their H-Ts all have the chance of “up-skilling” to better meet the needs of the population in the future. Aligning the incentives in the remuneration system for the dental workforce will play an important part in delivering these changes to create a service that is “fit-for-purpose” and based on quality.

## Competing interests

Funding to attend and present at the Prevention in Practice Conference was made available to Paul Brocklehurst by Colgate Palmolive who are also funding the publication of this manuscript. Richard Macey declares no competing interests.

## Authors’ contributions

Both authors made substantial contributions. PB and RM were responsible for drafting the document.

## Declarations

Funding to attend and present at the Prevention in Practice Conference was made available to Paul Brocklehurst by Colgate Palmolive who are also funding the publication of this manuscript.

This article has been published as part of *BMC Health Services Research* Volume 15 Supplement 1, 2015: Proceedings of Prevention in practice - making it happen. The full contents of the supplement are available online at http://www.biomedcentral.com/bmchealthservres/supplements/15/S1. Publication charges for this supplement were funded by Colgate Palmolive.
